# The Supporting Role of Hyperbaric Oxygen Therapy in Atopic Dermatitis Treatment

**DOI:** 10.3390/jcm14093138

**Published:** 2025-05-01

**Authors:** Michał Zwoliński, Adrian Hovagimyan, Jakub Ignatowicz, Marta Stelmasiak, Aneta Lewicka, Justyna Bień-Kalinowska, Barbara J. Bałan, Sławomir Lewicki

**Affiliations:** 1 Institute of Outcomes Research, Maria Sklodowska-Curie Medical Academy, Pl. Żelaznej Bramy 10, 00-136 Warsaw, Poland; michal.zwolinski@uczelniamedyczna.com.pl (M.Z.); justyna.bien-kalinowska@uczelniamedyczna.com.pl (J.B.-K.); 2 University Clinical Hospital in Opole, al. Witosa 26, 45-401 Opole, Poland; adrianhovagimyan8@gmail.com; 3 Faculty of Medical Sciences and Health Sciences, Casimir Pulaski University of Radom, Chrobrego 27 St., 26–600 Radom, Poland; panjakubignatowicz@gmail.com; 4 Department of Dietetics, Institute of Human Nutrition Science, Warsaw University of Life Sciences, Nowoursynowska 159c St., 02-776 Warsaw, Poland; marta_stelmasiak@sggw.edu.pl; 5 Military Centre of Preventive Medicine Modlin, 05-100 Nowy Dwór Mazowiecki, Poland; anet.lewicka@gmail.com; 6 Department of Environmental Threat Prevention, Allergology and Immunology, Faculty of Health Sciences, Medical University of Warsaw, Pawińskiego 3c, 02-106 Warsaw, Poland; barbara.balan@wum.edu.pl

**Keywords:** HBOT, immune system, immunomodulation, atopic dermatitis, supporting therapy

## Abstract

Over the past decades, atopic diseases have emerged as a growing global health concern. The Global Report on Atopic Dermatitis 2022 estimated that approximately 223 million people worldwide were living with atopic dermatitis in 2022, with around 43 million being children or adolescents. The financial burden associated with the treatment of this condition poses a significant challenge for both healthcare systems and patients. The current therapeutic approach for atopic diseases primarily focuses on symptomatic management, aiming to mitigate the effects of an overactive immune system. The most widely used treatments include topical or systemic corticosteroids, which suppress inflammation, and emollients, which help restore the skin barrier function. However, prolonged corticosteroid use is associated with adverse effects, including impaired immune response and reduced ability to combat external and internal threats. Consequently, there is a growing interest in developing alternative therapeutic strategies for managing atopic dermatitis. Among these emerging treatments, hyperbaric oxygen therapy (HBOT) appears particularly promising. HBOT has a beneficial effect on the vascular and immune systems, which results in improved functioning of tissues and organs. This therapy has demonstrated efficacy in promoting wound healing, particularly in conditions such as thermal burns and diabetic foot ulcers. Given these properties, HBOT is being tested as a potential adjunctive therapy for atopic dermatitis and other allergy-related diseases. In this paper, we present the current state of knowledge regarding the application of HBOT in the treatment of atopic and immune-mediated conditions, with a focus on its immunomodulatory and regenerative effects.

## 1. Introduction

Atopic dermatitis (AD), also known as atopic eczema, is a chronic, recurring inflammatory skin condition characterized by recurrent symptoms such as dry skin, itching, barrier skin dysfunction and characteristic eczematous lesions, and it is classified as an allergy. Typically, AD begins in childhood and sometimes may last into adulthood. Allergy is a clinical manifestation of an immune response to antigens, characterized by an excessive production of immunoglobulin E (IgE) [1]. Individuals with a genetic predisposition and/or inappropriate exposure to specific environmental factors are at an increased risk of developing hypersensitivity reactions to foreign antigens [2]. The increasing sanitization of living environments has contributed to reduced microbial exposure, particularly during early childhood. These disorders in the case of microbial contact may lead to alterations in the Th1/Th2 immune response balance, potentially increasing susceptibility to allergic diseases [3]. Globally, the prevalence of allergic diseases continues to rise. Epidemiological studies indicate that atopic dermatitis (AD), one of the most common allergic conditions, affects approximately 4.4% of adults and 18.6% of children in Europe [4]. In the United States, AD prevalence is reported at 7.2% among adults and 10.3% among adolescents aged 5 to 15 years. The prevalence is even higher in early childhood, the critical period during which AD symptoms typically manifest, affecting up to 25% of children aged 0 to 5 years [5,6]. The pathophysiology of allergic diseases is associated with the immune system’s inappropriate recognition of foreign antigens. The initial encounter with an allergen induces the sensitization phase, during which antigen-specific IgE antibodies are produced. Upon repeated exposure, activation of the effector phase occurs, leading to the release of inflammatory mediators and the onset of clinical symptoms.

## 2. Social Aspect

Atopic dermatitis (AD) is a chronic and recurrent inflammatory skin disorder characterized by pruritus, oedema, and eczematous skin lesions. Most epidemiological data on AD stem from studies focusing on the paediatric population. According to the Global Burden of Disease Study, AD affects approximately 15–20% of children, whereas its global prevalence among adults, ranges from 2.1% to 10% [7,8]. The prevalence of AD varies significantly across different countries. In Poland, the Epidemiology of Allergic Diseases in Poland study estimated an AD prevalence of 6.5%, with the highest occurrence reported among women and individuals living in major urban areas [9].

A loss-of-function mutation in the filaggrin gene disrupts skin barrier function, increasing susceptibility to AD. Atopic dermatitis (AD) can be classified into three types based on the age of onset: infantile, paediatric, and adult. While the core clinical features of AD—such as xerosis, flexural area involvement, and atopic predisposition—are generally consistent across age groups, disease expression varies. In adults, AD tends to present with more chronic symptoms, a higher prevalence of hand eczema, and a stronger correlation between disease activity and emotional factors. In contrast, children more commonly exhibit a seborrheic dermatitis-like presentation [10].

Atopic dermatitis (AD) is a chronic condition with no definitive cure. Persistent pruritus and eczema significantly impact daily activities and sleep and impair patients’ quality of life. Additionally, individuals with AD often experience social stigma associated with visible skin symptoms, contributing to a higher prevalence of depression in this group [11]. The economic expense of AD affects both patients and healthcare systems. A 2013 study in Spain estimated the average annual outlay of AD, including healthcare burden and lost workdays, at EUR 1504 per patient. Costs escalated with disease severity, reaching up to EUR 3686 for severe cases. Of this total, 75.5% was attributed to treatment expenditures, while 24.5% resulted from productivity losses due to absenteeism or reduced work efficiency [12]. In a study by Zuberbier et al., AD patients experienced an average of nine exacerbations per year, each lasting approximately 15 days. This resulted in an average of 2.5 days per year spent absent from school or work, with an additional 9% reduction in productivity due to impaired concentration caused by disease symptoms [13]. Moreover, patients with severe AD experienced an average of 136 days of exacerbations per year, which, in turn, led to an increased number of work absences. [13]. In addition to its financial expenses on healthcare systems, atopic dermatitis (AD) also exerts a significant economic strain on the personal budgets of affected individuals. In 2017, the average out-of-pocket expenditure for a European patient was EUR 927.12, with the largest proportion (35.7%) allocated to the purchase of emollients [14].

## 3. Pathomechanism

The development of atopic dermatitis (AD) is influenced by a complex interplay of genetic, environmental, and immune-related factors. The pathophysiology of AD is primarily characterized by immune system dysregulation, with a shift towards a predominant Th2 response. This immune imbalance contributes to intense pruritus, erythema, and impaired skin barrier function. Structural abnormalities in the skin involve both altered lipid composition and disrupted architectural organization, leading to reduced barrier efficacy, increased transepidermal water loss, xerosis, and an elevated skin pH. These changes facilitate the penetration of pathogens through the epidermal barrier, triggering immune activation via Langerhans cells, dendritic cells, and T cells, as well as immunologically active keratinocytes, which produce pro-inflammatory mediators, ultimately resulting in chronic inflammation [15]. This chronic inflammatory state promotes the activation of antigen-presenting cells (APCs) and the release of thymic stromal lymphopoietin (TSLP), a cytokine that amplifies the Th2 response [16,17]. Consequently, this cascade of immune activation can drive the transition from a non-atopic to an atopic phenotype via IgE sensitization [18,19]. Furthermore, bacterial infections have been shown to exacerbate AD by inducing pro-inflammatory responses through Toll-like receptor 2 (TLR2), leading to the upregulation of the high-affinity IgE receptor (FcεRI) on dendritic cells in AD patients [20]. This enhances antigen capture by Langerhans and dendritic cells, improving antigen presentation to T cells and further propagating the allergic response [21]. Additionally, the simultaneous activation of TLRs and IgE receptors on basophils stimulates the production of IL-4, IL-8, and IL-13, promoting the differentiation of naïve CD4+ T cells into the Th2 lineage [20,22]. Recent studies have identified the presence of IgE-expressing dendritic cells, including APCs, inflammatory dendritic epidermal cells, and Langerhans cells, in the lesional epidermis of patients with IgE-allergic AD. These cells capture environmental allergens, such as house dust mites, contributing to chronic immune activation in lichenified eczema [23].

Among the cytokines involved in AD pathogenesis, IL-4 plays a fundamental role by strongly stimulating B lymphocytes and promoting class switching towards IgE production. Additionally, through positive feedback mechanisms, IL-4 enhances Th0 differentiation into Th2 cells, leading to increased production of IL-4, IL-5, and IL-13 [24]. IL-4 influences multiple immune cell populations, often exerting opposing effects, and induces the secretion of chemokines (CCL3L1, CCL8, CCL24, CCL25, CCL26, CXCL6, CXCL16), which recruit additional immune cells and prolong inflammation [25]. Elevated levels of IL-5 and eosinotaxins (primarily CCL11, CCL25, and CCL26, induced by IL-25) are also observed in AD patients. These molecules promote eosinophil migration to inflamed skin, where they degranulate and release cytotoxic mediators, exacerbating tissue damage [26,27]. Additionally, increased levels of TL1A (TNF-like ligand A) have been linked to the promotion of inflammatory responses and the expansion of Th2 and Th17 lymphocytes [28]. Notably, Th17 cell infiltration is also elevated in AD, as observed by Chovatiya and Silverberg [26]. Th17 cells secrete IL-17A, IL-17F, IL-22, and tumour necrosis factor-alpha (TNF-α), further contributing to skin inflammation and barrier dysfunction [29]. Interestingly, our research group was the first to demonstrate that keratinocytes isolated from AD patients are capable of synthesizing IL-17, suggesting that Th17 lymphocytes are not the sole contributors to IL-17-mediated inflammation in AD [30]. The crucial role of IL-4 in AD pathogenesis is further supported by the clinical efficacy of dupilumab, an IL-4/IL-13-targeting biologic, which has been shown to significantly improve disease outcomes in both IgE-allergic and non-IgE-allergic AD phenotypes [31]. Moreover, IL-4, through interaction with IL-4RA, as well as IL-13 and IL-31, induces phosphorylation of the JAK1 pathway, thereby enhancing neuronal responsiveness to multiple pruritogens by activating sensory neuron receptors responsible for transmitting pruritus signals to the central nervous system [32,33].

**Figure 1:**
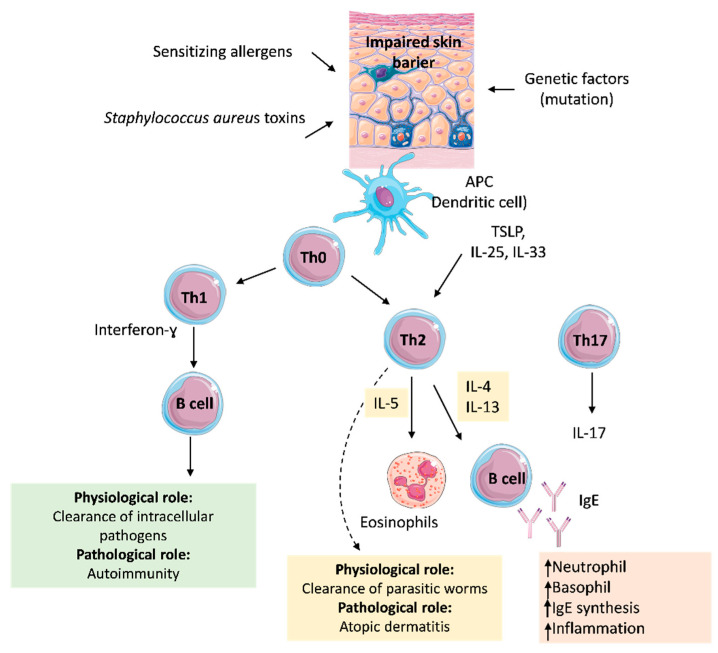
A simplified schematic representation of the pathogenesis of atopic dermatitis (AD) characterized by a compromised skin barrier. The influence of infections, allergens, and genetic predisposition contributes to the upregulation of inflammatory signalling pathways. ↑—increase.

The immune response mechanism involved in atopic dermatitis (AD) is presented in Figure 1.

## 4. Concomitant Infections

Skin inflammation caused by the infiltration of immune system cells changes the structure of the epidermis and causes skin scratching [34]. Both factors increase the risk of infection. Patients with atopic dermatitis (AD) exhibit a heightened susceptibility to recurrent skin infections caused by bacteria (*Staphylococcus aureus* and *β-haemolytic Streptococcus pyogenes*), viruses (*herpes simplex virus*, HSV), and fungi (*Malassezia* spp. and *Candida* spp.). The most common skin infections in AD inflammation are caused by *S. aureus*; this bacterium is present in 93% of AD patients. The *S. pyogenes* bacterium is the second most frequent cause of skin infections in AD lesions [35]. The pathogenesis of bacterial infections in AD involves bacterial virulence factors, including toxins (e.g., staphylococcal enterotoxins), enzymes, and bacterial cell wall components. These factors exacerbate skin inflammation and contribute to bacterial persistence, epithelial penetration, and infection. The *S. aureus* bacterium has been shown to induce the release of thymic stromal lymphopoietin (TSLP), interleukin (IL)-31, and IL-33 from keratinocytes in AD patients [36,37,38,39]. These cytokines establish a positive feedback loop that aggravates AD symptoms, such as pruritus, erythema, and skin barrier dysfunction, while also promoting the recruitment of eosinophils and mast cells, thereby amplifying the inflammatory response. Notably, a positive correlation has been observed between the severity of inflammation and the extent of *S. aureus* colonization, whereas *Staphylococcus epidermidis* does not exhibit a similar association. Clinically, *S. aureus*-induced skin infections in AD patients are characterized by weeping lesions, honey-coloured crusts, and pustules. Although pustules are an uncommon feature of AD, their presence may be associated with significant pruritus and pain [40].

Atopic dermatitis (AD) patients are also predisposed to viral skin infections, though these occur less frequently than bacterial infections. Viral infections include eczema herpeticum, eczema coxsackium, eczema vaccinatum, and molluscum contagiosum, with eczema herpeticum (EH) being one of the most common among AD patients [35]. The pathogenesis of EH in AD involves genetic variations in innate immune response molecules, including TSLP and interferons (IFN-α, β, γ, and ω), as well as activation of the STAT6 gene, which enhances viral replication [41]. Eczema herpeticum (EH) is caused by HSV (*Herpes simplex virus*) infection and represents a potentially life-threatening complication in AD patients. The lesions predominantly affect the face, neck, upper trunk, and antecubital/popliteal regions. Clinically, viral infections in AD manifest as pruritus or pain, vesicles, punched-out erosions, or haemorrhagic crusts. Localized skin infections may progress to disseminated vesicles with skin barrier disruption. Systemic EH may present with fever, malaise, viremia, and severe complications such as keratoconjunctivitis, encephalitis, and septic shock [42].

Fungal infections are also common in AD patients, particularly those caused by *Malassezia* spp., which are part of the normal human cutaneous microbiota. Impairment of the skin barrier facilitates immune system exposure to these yeasts [43]. *Malassezia* colonization induces inflammation in AD patients, particularly in sebaceous gland-rich regions such as the head, neck, upper chest, and back. In some cases, recurrent *Malassezia* infections may trigger IgE-mediated autoreactivity via molecular mimicry between fungal allergens and homologous human proteins, perpetuating chronic skin inflammation [44]. *Candida* spp. are significant fungal colonizers of mucosal surfaces, including the genitourinary tract, oral cavity, and gastrointestinal tract. While data on *Candida* colonization in AD patients remain limited, *C. albicans* has been associated with severe infections in immunocompromised individuals [43]. Specific protein components of *C. albicans* (27, 37, 43, 46, 125, and 175 kDa) may play a role in AD pathogenesis by stimulating immune responses and interacting with immunoglobulins. Additionally, increased AD severity has been correlated with elevated *C. albicans*-specific antibody production [45].

Microbiological infections are a prevalent complication in atopic dermatitis (AD) and significantly influence both their frequency and duration. Effective management requires the use of local and systemic antimicrobial therapy, which not only increase treatment costs but also compromise the skin’s protective barrier by reducing the population of commensal microorganisms [46].

## 5. Conventional Treatment

The treatment of atopic dermatitis (AD) falls within the domain of dermatology. Conventional therapies primarily include topical medications, systemic treatments, proper skin care, phototherapy, immunomodulatory therapy, and lifestyle modifications. In many cases, a combination of these therapeutic approaches proves effective; however, long-term treatment is often required and may be associated with adverse effects. Topical medications, such as corticosteroids and calcineurin inhibitors, serve as first-line treatments, alleviating inflammation and pruritus. However, prolonged use can lead to adverse effects, including skin atrophy, telangiectasia (dilation of small blood vessels), and localized irritation. Proper skin care, which will be discussed in detail later, requires a high level of patient adherence and cooperation. Phototherapy, while effective, necessitates specialized equipment, precise dosing, and carries a potential risk of skin cancer. Currently, only a limited number of immunomodulatory therapies, particularly immunosuppressive treatments, are approved for AD management. These therapies demonstrate efficacy in a subset of patients but are not without adverse effects. Lifestyle modifications, including allergen avoidance and stress reduction, play a role in disease management; however, their impact is challenging to quantify.

In 2015, the estimated annual cost of AD treatment in the United States exceeded $5 billion. More recent studies indicate that patients with moderate-to-severe AD incur significantly higher treatment costs compared with those with mild disease, primarily due to the increasing use of biologic therapies [47,48]. Additionally, in Japan, the cost of AD drug treatment in 2011 amounted to JPY 637,526,063 [49].

### 5.1. Emollients

The primary goal of atopic dermatitis (AD) treatment is to restore the damaged skin barrier. Proper AD skin care, a cornerstone of AD management, involves the regular and appropriate application of emollients to the affected skin [50]. Emollient therapy is widely recognized for its role in reinforcing and maintaining the integrity of the *stratum corneum*, potentially reducing the need for topical glucocorticoids [51]. Some studies suggest that regular emollient usage may prevent the onset of AD in genetically predisposed individuals, though findings remain inconclusive [52]. Emollients constitute the cornerstone of AD management and should be applied 2–3 times per day, with an estimated weekly usage of 250–500 g [53]. The frequent application required for effective management contributes to the financial burden on patients. A 2019 study conducted in France estimated that the average cost of emollients per patient amounted to approximately EUR 750 over five years, comparable to the total healthcare expenses associated with medical consultations and hospitalizations [54]. However, consistent emollient use has been shown to significantly reduce the frequency of medical visits and the need for antibiotics, suggesting an overall improvement in patients’ quality of life [55]. The research shows that cosmetics with addition of selected species probiotics can be useful too. For example, *Lactobacillus johnsonii* NCC 533, can inhibit *Staphylococcus aureus* adherence to the keratinocytes—even after heat treatment [56].

### 5.2. Glucocorticoids and Immunosuppressants

In 2024, the American Academy of Allergy, Asthma, and Immunology and the American College of Allergy, Asthma, and Immunology Joint Task Force released updated guidelines incorporating new evidence and treatment options for AD [57]. Despite emerging therapies, conventional pharmacological treatment remains the primary approach. Depending on AD severity, either topical or systemic anti-inflammatory treatments are administered. Topical corticosteroids represent first-line therapy in most cases. Initially, potent formulations are prescribed to achieve symptom control, followed by milder variants to maintain remission. In mild-to-moderate AD, topical treatment options include tacrolimus, a calcineurin inhibitor. Tacrolimus exerts its effects by reducing the expression of the Fc receptor on Langerhans cells and inhibiting their activation of T lymphocytes. Additionally, tacrolimus blocks calcium-dependent signal transduction pathways in T lymphocytes, thereby preventing the transcription and synthesis of interleukins IL-2, IL-3, IL-4, IL-5, as well as other cytokines such as granulocyte-macrophage colony-stimulating factor (GM-CSF), tumour necrosis factor-alpha (TNF-α), and interferon-gamma (IFN-γ) [58]. For moderate-to-severe AD, systemic immunosuppressants such as cyclosporine and tacrolimus are often required. In certain cases, systemic antihistamines are also included in the treatment regimen [59].

### 5.3. Biological Treatment

In cases of moderate-to-severe AD, systemic therapies including biological treatment are often necessary. The FDA has approved these four biologics to treat atopic dermatitis (AD): dupilumab, lebrikizumab, nemolizumab, and tralokinumab. Dupilumab is a monoclonal antibody targeting the IL-4α receptor, thereby modulating the inflammatory response [60]. There are also attempts to block the receptor OX40 (CD134) and OX40L (CD252) (expressed on activated CD4 and CD8 cells) by antibodies (still in clinical trials) [61]. Anti-OX40 antibody (rocatinlimab) inhibits one of the main T cell differentiation pathways, reducing the severity of AD even after the end of the treatment [62]. Other clinical trials concern JAK inhibitors, like tofacitinib and baricitinib, which modulate immune responses by interfering with cytokine signalling pathways, leading to reduced inflammation and itching symptom relief [63]. However, the use of biological treatment significantly increases the costs of treatment [46]. A study by Ong et al. demonstrated that the monoclonal antibody dupilumab, compared with standard therapy, increased the cost of treatment by as much as 4.88 times. Moreover, biological treatment has many side effects [64].

## 6. Hyperbaric Oxygen Therapy

Hyperbaric oxygen therapy (HBOT) is a therapeutic method that uses almost 100% oxygen concentration at pressures of at least 1.4 atmospheres (ATMs). However, all currently approved applications require at least 2.0 ATMs. Typically, pressures no higher than 3.0 ATMs are used due to side effects occurring above this value [65]. Hyperbaric oxygen therapy (HBOT) causes a significant increase in oxygen concentration in the blood serum and tissues including subcutaneous tissue oxygenation. This is believed to cause oxidative stress with an increase in reactive oxygen species (ROS) and nitric oxide synthase activity [66,67]. It is generally accepted that free radicals are harmful to the organism, but studies have not shown any harmful effects on DNA and proteins [68,69]. HBOT has become a popular treatment option for a range of medical disorders. HBOT was first used to treat pathophysiological disorders in divers and mine workers, but it has now been expanded to include effective treatment of wounds, thermal or radiation burns, and CO poisoning [70]. This method is also used to treat gas embolism, severe anaemia, gas gangrene, crush injuries/compartment syndrome/acute traumatic ischemia, decompression sickness, arterial inefficiencies, intracranial abscesses, necrotizing soft tissue infections, osteomyelitis, and idiopathic sensorineural hearing loss [71,72,73].

One of the common uses of HBOT is the treatment of thermal burns. In studies on animal models, it has been noted that the use of this therapy reduces tissue oedema because of vasoconstriction and increases proliferation of cells involved in regeneration processes. However, this does not cause hypoxia due to the increased oxygen-carrying capacity of the blood [74]. HBOT reduces the formation of thrombosis within the damaged tissue, while reducing the adhesion of neutrophils to the endothelium, which prevents damage to the microcirculation and allows blood to flow to the burnt area [75]. It also accelerates restoration of the epidermis continuity [76]. The use of HBOT causes faster healing of skin lesions and a reduction in the severity of histological changes. Studies in volunteers and patients have shown a reduction in exudate and redness, as well as a reduction in wound size [75]. Similar positive effects of the use of HBOT are observed in diabetic foot patients. Meta-analyses of previous studies have found a statistically significant reduction in the time needed to heal [77], including an increased rate of complete ulcer healing [78]. Recent studies demonstrated the beneficial effect on skin graft acceptance and regeneration after transplantation [79]. HBOT also plays a function in vascularization, angiogenesis, and collagen production augmentation [80].

**Figure 2:**
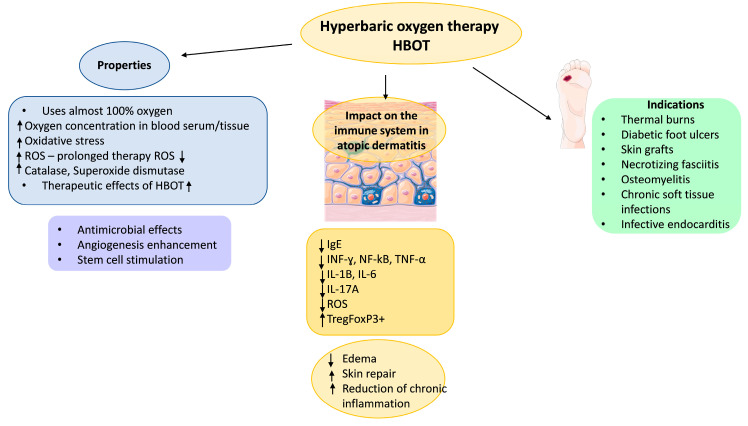
The beneficial impacts of hyperbaric oxygen therapy (HBOT) on atopic dermatitis (AD). Summary of the properties of HBOT and its clinical applications. ↑—increase, ↓—decrease.

Initially, HBOT causes an increase in the level of ROS by deleterious effect on the mitochondria, but with prolonged therapy, the level of ROS decreases. The influence of HBOT on the increase in the activity of antioxidant enzymes, especially catalase and superoxide dismutase, is suspected. Some researchers consider the increase in the activity of antioxidant enzymes to be an important element of the therapeutic effect of hyperbaric oxygenation [81,82,83,84]. The effect of HBOT on the immune system is currently very poorly understood. Clinical observations indicate the anti-inflammatory effect and promotion of tissue regeneration. Several studies have shown a marked reduction in C-reactive protein (CRP) as well as pro-inflammatory cytokines such as interferon-gamma (IFN-γ) [85], nuclear factor-kappa B (NF-κB) [86,87] and tissue necrosis factor-alpha (TNF-α) [88,89,90]. Some authors suggest a decreasing effect on the levels of pro-inflammatory IL-1, IL-1β, and IL-6 [91,92,93]. On the contrary, HBOT enhanced the production of anti-inflammatory mediators, i.e., IL-10 [94]. Additionally, HBOT treatment in volunteers reduced ROS production, and a diminished phagocytosis capacity by neutrophils was noted despite increased oxygen availability [95]. Considering the described aspects of health improvement, HBOT is a promising tool for possible use in allergic diseases. The beneficial effects of hyperbaric oxygen therapy (HBOT) on atopic dermatitis (AD) are presented in Figure 2.

## 7. Treatment of Atopic Dermatitis with HBOT

Studies on the effect of HBOT on the immune system in atopic dermatitis (AD) remain very scarce. Generally, HBOT is known for its healing effects on the skin. Increased oxygen delivery facilitates essential biological processes such as cellular proliferation and collagen synthesis, which helps to repair the architecture of pathologically damaged skin and restore its continuity and barrier function [96]. HBOT results not only in a temporary increase in tissue oxygen saturation but also in an increase in VEGF expression in endothelial cells, fibroblasts, keratinocytes, and immune cells, which promotes angiogenesis in the skin, contributes to improved vascularization and better tissue oxygenation in the long term [97]. Another research study proved that HBOT, via increasing ROS and NO, can activate endothelial progenitor cells, which are essential for neovascularization—which additionally enhances the wound healing process [98].

A particularly pronounced therapeutic effect is also reflected in the reduction of total serum IgE concentration [74,99,100,101,102]. The study conducted by Mews et al. on children with atopic dermatitis (AD) found no statistically significant impact of hyperbaric oxygen therapy (HBOT) on the concentrations of selected interleukins (IL-4, IL-6, and IL-10) or the percentage distribution of regulatory T (Treg) lymphocytes, which play a crucial role in suppressing the inflammatory response. However, clinical symptoms of the disease were significantly alleviated after 30 days of HBOT, as assessed using the SCORAD and oSCORAD scales [99]. Romuald Olszański et al. conducted two clinical studies investigating the efficacy of hyperbaric oxygen therapy (HBOT) in treating atopic dermatitis (AD). In the first study, carried out in 1992, five patients diagnosed with AD underwent daily HBOT sessions at a pressure of 0.1 MPa over 15 days [101]. All participants exhibited clinical improvement, accompanied by a reduction in serum immunoglobulin E (IgE) levels and complement components. A subsequent study in 2017 involved ten patients with severe, treatment-refractory AD [102]. Participants received ten HBOT sessions at 2.5 atmospheres absolute (ATA) across two weeks. Notably, all subjects demonstrated clinical improvement, including a marked reduction in pruritus, despite suspending systemic therapy. Immunological assessments conducted before and after HBOT revealed a consistent downward trend in the concentrations of immunoglobulins and complement components, with the most notable reductions observed in IgE and complement C3 levels. These findings collectively support the hypothesis that HBOT may exert beneficial immunomodulatory effects in patients with AD, potentially influencing the disease’s clinical course.

Topical applications of ozone and oxygen-based therapies have also been investigated for their therapeutic potential in the management of atopic dermatitis (AD) and related inflammatory dermatoses. Bennardo et al. conducted a clinical study utilizing oxygen infusion therapy, a technique in which pure oxygen is delivered to the superficial layers of the skin, facilitating its diffusion into the dermis. In this study, twenty-four patients with AD underwent twelve treatment sessions, resulting in a mean SCORAD (Scoring Atopic Dermatitis) reduction from 3.6 to 2.1—an average decrease of 1.5 points. Notably, these clinical improvements were sustained at follow-ups conducted at one- and three-month post-treatment visits [103]. In a separate investigation, Lu et al. [104] evaluated the efficacy of topical ozonated formulations in twelve patients with moderate-to-severe AD. The study employed a split-body design, wherein symmetrical lesions were assigned to either treatment or control conditions. The treatment sites were washed with ozonated water and treated with ozonated oil over a period of seven days, while control sites received tap water and base oil. The treatment group demonstrated significant clinical improvement, including a reduction in pruritus severity and a statistically significant enhancement in SCORAD scores compared with the controls (*p* < 0.01). Confocal microscopy analysis revealed a reduction in parakeratosis and inflammatory cell infiltration. Moreover, the treatment group exhibited a significant decrease in *Staphylococcus aureus* colonization (*p* < 0.01), with a strong linear correlation observed between the reduction in bacterial load and SCORAD score improvement (*p* = 0.001). Further evidence supporting the role of oxygen-based topical interventions was provided by Doğan and Dinç Kaya [105], who explored the use of topical oxygen therapy in infants diagnosed with diaper dermatitis. In this study, thirty infants received topical oxygen exposure to inflamed areas for one hour following each diaper change. The intervention led to a notable reduction in both symptom severity and overall recovery time, underscoring the potential utility of oxygen therapy in paediatric inflammatory skin conditions A comprehensive summary of studies evaluating hyperbaric oxygen therapy (HBOT) and oxygen treatment in patients with atopic dermatitis (AD) is presented in Table 1.

In a murine model of atopic dermatitis (AD), Kim et al. observed an increase in reactive oxygen species (ROS) levels, accompanied by a simultaneous decrease in the pro-inflammatory cytokines IL-17A and IFN-γ, which play a crucial role in the chronic phase of AD. Additionally, a significant reduction in hypoxia-inducible factor 1-alpha (HIF-1α) was noted in the HBOT-treated group compared with the untreated AD group. Simultaneously, a substantial increase in the levels of indoleamine 2,3-dioxygenase (IDO) was detected. A statistically significant rise in the number of anti-inflammatory Treg FoxP3+ lymphocytes was also observed in the treatment group. Although IDO is part of the interferon signalling pathway, its concentration increased despite a marked reduction in IFN-γ levels [74]. HIF-1α is known to promote Th17 lymphocytes while inhibiting the differentiation of Treg cells; thus, its downregulation may have a beneficial effect in the treatment of autoimmune diseases [106]. It is hypothesized that the effects of hyperoxia on these factors contribute significantly to shifting the immune environment from a pro-inflammatory to an anti-inflammatory state. Furthermore, Kim et al. reported a reduction in the severity of histological changes, comparable to the effects observed in the group receiving topical corticosteroids. The severity of oedema, measured by epidermal thickness, and the infiltration of inflammatory cells within the tissue, including mast cells (which release histamine in response to IgE stimulation or cellular damage), were notably reduced [74].

The growing body of evidence supporting the efficacy of hyperbaric oxygen therapy (HBOT) in the management of atopic dermatitis (AD) has led to its increasing consideration as a novel adjunctive treatment [107]. For cases of treatment-resistant eczema, HBOT may serve as a valuable therapeutic option. The increasing popularity of oxygen therapy for a range of medical disorders [80] and within the field of aesthetic medicine [108,109] has contributed to a reduction in procedural costs and to the increased development of portable devices, enabling treatments to be conducted outside hospital settings, including at home or in beauty salons. The accessibility of such therapeutic support could significantly improve patient access to symptom relief, potentially reducing the severity and frequency of AD flares or lowering the required dosages of pharmacological treatments. Consequently, this could lead to a substantial improvement in the overall quality of life for individuals with AD. It is also worth noting that hyperbaric oxygen therapy (HBOT) can potentially reduce treatment costs for both patients and healthcare systems, as demonstrated in the case of diabetic foot ulcer management [110]. The higher direct costs of HBOT are compensated for by the less frequent need for control visits [111]. However, the cost-effectiveness of hyperbaric oxygen therapy (HBOT) remains uncertain, as some studies have failed to demonstrate a statistically significant difference between HBOT and standard therapy, while others have even suggested a cost disadvantage. A study by Brouwer RJ et al. found that HBOT, when used as an adjunct to standard therapy in patients with ischemic diabetic foot ulcers, did not result in significant differences in cost or health benefits [112]. Similarly, a study by Mindrup et al. calculated that HBOT treatment incurs higher costs for hospitals, even before accounting for subsequent outpatient care. This limitation complicates the assessment of the economic value of HBOT as a treatment option [113].

It is important also to acknowledge that HBOT may, in some rare cases, lead to adverse effects. The most common complications associated with this therapy include claustrophobia and barotrauma [114]. Additionally, ocular complications such as hyperbaric myopia, cataract formation, keratoconus, and retinopathy of prematurity have been reported. Patients with pre-existing respiratory conditions, including chronic obstructive pulmonary disease (COPD), asthma, or upper respiratory infections, may be at increased risk of complications during HBOT [115]. Furthermore, certain physiological and medical conditions, such as pregnancy [116], hypoglycaemia, epilepsy, hyperthyroidism, and the concurrent use of specific medications, may serve as potential contraindications to HBOT [117].

## 8. Conclusions

Hyperbaric oxygen therapy (HBOT) demonstrates potential as an adjunctive treatment for atopic dermatitis (AD) by modulating inflammation and promoting tissue regeneration. In atopic dermatitis (AD), HBOT may facilitate skin repair by enhancing oxygen delivery to hypoxic tissues and mitigating chronic inflammatory processes. Additionally, its intrinsic antimicrobial properties could contribute to the prevention or resolution of secondary infections commonly associated with this condition. HBOT has also been shown to influence immune regulation, leading to a reduction in allergic and inflammatory symptoms. Moreover, emerging evidence suggests that HBOT may alter cytokine profiles, shifting the immune response from a pro-inflammatory to an anti-inflammatory state, which could be beneficial in chronic dermatological conditions. Its potential to downregulate hypoxia-inducible factor 1-alpha (HIF-1α) and promote regulatory T cell differentiation further supports its immunomodulatory role. However, despite these promising findings, further large-scale clinical trials are essential to validate its therapeutic efficacy, optimize treatment protocols, and establish safety guidelines for its clinical application in atopic dermatitis (AD).

## Figures and Tables

**Table 1:** Summarized up-to-date effect of oxygen therapy in humans. We searched three main databases with articles: PubMed, Google Scholar, and SCOPUS with queries: HBOT and atopic dermatitis; HBOT and eczema; Hyperbaric and atopic dermatitis; or oxygen therapy and atopic dermatitis. We present here the results from only original revised articles. AD—atopic dermatitis; SCORAD—SCORing Atopic Dermatitis, ATA—absolute atmospheres.

**Hyperbaric Oxygen Therapy (HBOT)**			
**Authors**	**Group of Patients**	**Treatment**	**Significant Observations**
Mews et al., 2021 [99]	15 children (3–18 years) with severe AD (SCORAD > 50)	2.5 ATA, (~250 kPa), 30-day treatment	clinical improvement (reduction in the intensity of pruritus, improvement in sleep quality);
			significant decrease of IgE in the serum
Olszański et al., 1992 [101]	5 patients (8–38 years) with atopic dermatitis	0.1 MPa pure oxygen, 15 days of treatment	clinical improvement;
			significant decrease of IgE and the level of C3 and C4 complement
Olszański et al., 2017 [102]	10 adult patients (18–44 years) with severe atopic dermatitis who did not respond to standard pharmacotherapy	10 oxygen exposures at pO_2_ 2.5 ATA each	clinical improvement (local improvement in the dermatological condition, reduction in the experienced itch, reduction in the use of oral antipruritic drugs, i.e., antihistaminic and/or hydroxyzine);
			significant decrease of IgE and the level of C3 complement
**Topical Oxygen Therapy**			
Bennardo et al., 2018 [103]	24 adult patients with atopic dermatitis (mean age 34 years) with an initial SCORAD disease severity index between 2 and 6.	Ttopical oxygen therapy, 3 sessions of oxygen therapy per week, in total—12 sessions	clinical improvement (decreased SCORAD disease severity index for patients with eczema, and atopic dermatitis showed a decline (the mean score dropped from 3.6 to 2.1 after 12 sessions)
Lu et al., 2018 [104]	12 patients (6–65 years) with moderate or severe AD	ozonated water and smeared with ozonated oil, 7 days of treatment	clinical improvement (decreased SCORAD scores, and pruritus scores, improvementing in sleep quality); a linear correlation between the decreasing percentage of S. aureus *S. aureus* colony and the declining percentage of SCORAD scores in AD patients
Doğan and Dinç Kaya, 2024 [105]	30 patients (newborns) with diaper dermatitis	oxygen applied for 1 h at each diaper change at a flow rate of 5 L/min and at a concentration of 21% FiO_2_, which is equivalent to room air.	clinical improvement (administration of oxygen to the diaper dermatitis area reduceds the severity and shorteneds the recovery time of diaper dermatitis)

## Data Availability

Not applicable.

## Abbreviations

The following abbreviations are used in this manuscript:

HBOT	hyperbaric oxygen therapy
AD	atopic dermatitis
IgE	immunoglobulin E
APCs	antigen-presenting cells
TSLP	thymic stromal lymphopoietin
IL	interleukine
TLR	Toll-like receptor
EH	eczema herpeticum
IFN	interferon
TNF	tumour necrosis factor-alpha
JAK	Janus kinase
ROS	reactive oxygen species
SCORAD	scoring atopic dermatitis
oSCORAD	objective component of SCORAD
HIF-1α	hypoxia-inducible factor 1-alpha
